# Redox Signaling and the Cardiovascular and Skeletal Muscle System

**DOI:** 10.1155/2015/849095

**Published:** 2015-10-07

**Authors:** Aldrin V. Gomes, Namakkal S. Rajasekaran, Xinchun Pi

**Affiliations:** ^1^Department of Neurobiology, Physiology, and Behavior, University of California, Davis, CA 95616, USA; ^2^Cardiac Aging & Redox Signaling Laboratory, Division of Molecular & Cellular Pathology, Department of Pathology, University of Alabama at Birmingham, Birmingham, AL 35294, USA; ^3^Division of Cardiovascular Medicine, Department of Medicine, University of Utah School of Medicine, Salt Lake City, UT 84132, USA; ^4^Department of Medicine, Cardiovascular Research Institute, Baylor College of Medicine, Houston, TX 77030, USA

The role of reactive oxygen species (ROS) in skeletal, cardiac, and vascular dysfunctions has been under investigation for decades. Over the last decade advanced* in vitro* and* in vivo* approaches have significantly improved our understanding of the role of oxidative stress in cardiovascular and skeletal muscle diseases. Oxidative stress is regarded as an imbalance between the production of free radicals/ROS and the cell's intrinsic antioxidant defense mechanisms. Overburden of oxidative stress has been associated with atherosclerosis, ischemic heart disease, hypertension, cardiomyopathies, cardiac hypertrophy, heart failure, and skeletal muscle diseases such as muscular dystrophy and sarcopenia. This special issue provides insights into understanding of the role of oxidative stress as a potential causal mechanism in various forms of cardiovascular and skeletal disease models and possible protection through antioxidant supplementation.

The original research article by T. L. Lynch et al. demonstrates for the first time that oxidative stress is elevated in a cardiac myosin binding protein C mouse model of dilated cardiomyopathy (DCM). DCM heart homogenates show a lower reduced/oxidized glutathione (GSH/GSSG) ratio, increased oxidized (carbonylated) proteins, increased lipid peroxidation, and reduced signals for mitochondrial semiquinone radicals and Fe-S clusters (as measured by electron paramagnetic resonance spectroscopy) compared to healthy control heart homogenates. Similar results were obtained when human cardiomyopathy heart homogenate samples were investigated. These results strongly suggest that oxidative stress may exacerbate the development of heart failure.

In the original study by L. A. Moreno-Ruíz et. al., the authors demonstrate the effects of oxidative stress on left ventricular mechanics in hypertensive patients. These studies highlight the significance and contribution of oxidative stress in hypertensive patients with diastolic dysfunction and preserved ejection fraction. Heart failure with preserved ejection fraction is an emerging clinical condition in a wide range of cardiac patients that warrants further mechanistic understanding. The authors showed that a significant increase in the levels of oxidized proteins coupled with impaired antioxidant defenses in essential hypertensive patients might be a potential cause for cardiac dysfunction. The original work performed by A. Gawron-Skarbek et al. demonstrates an interesting observation that physically active and fit male patients with coronary heart disease (CHD), whilst having a more favorable cardiometabolic diseases risk profile, are characterized by lowered antioxidant capacities. This manuscript also discovered that uric acid was not only a potent antioxidant but also a cardiovascular risk factor. These results provide insights for future planning of effective cardiac rehabilitation in CHD patients.

The review by R. Ghosh et al. focuses on the role of nonsteroidal anti-inflammatory drugs (NSAIDs) in cardiovascular disease. NSAIDs are one of the most commonly used drugs worldwide and have been associated with increased risk of cardiovascular disease. The authors present results to support their suggestion that the cardiovascular effects may be partly due to high ROS levels produced by NSAIDs. Another review by P. Angsutararux et al. outlined oxidative stress as a trigger/causative mechanism for chemotherapy-induced cardiotoxicity. The studies connecting chemotherapy and cardiotoxicity have attracted the field of cardiovascular research within the past decade. The authors elegantly outlined the key published findings of the effects of different cancer drugs that lead to structural and functional remodeling of the heart. Importantly, this review updates what is currently known about the cross talk between chemotherapy, oxidative stress, and cardiotoxicity. ROS has been previously shown to affect the function of oxygen sensing proteins. The review by W. H. D. Townley-Tilson et al. is focused on the crucial role of oxygen sensing proteins in heart function and ischemic heart disease. In particular, the review elaborates the recent exciting progress about prolyl hydroxylase domain proteins (PHDs) in the regulation of cardiac homeostasis.

Muscle wasting occurs due to aging or as consequence of many diseases including chronic kidney disease (CKD). The original research by D.-T. Wang et al. shows that CKD upregulated the expression of myostatin and associated skeletal muscle breakdown through activation of both autophagy and the ubiquitin-proteasome system (UPS). These molecular events are mechanistically linked to the PI3K/Akt/FoxO3a signaling pathway. The article by A. Felipe et al. shows convincing evidence from a group of 66 Chilean male volunteers indicating that serum levels of ferritin, a protein that regulates iron homeostasis, are associated with metabolic syndrome and serve as a clinical or diagnostic marker for chronic diseases that are related to oxidative stress, hepatic damage, insulin resistance, and renal insufficiency. Of interest, this study identified a strong positive association between red meat consumption and serum ferritin.

Selenium (Se) is an exogenous antioxidant that performs its function by stimulating the expression of selenoproteins such as glutathione peroxidases (GPx) in endothelial cells. The original article by B. Ruseva et al. demonstrates that Se supplementation increases GPx-1 activity and suppressed lipid peroxidation in rats. These results suggest that Se supplementation may be a potential therapeutic and preventive strategy against vascular disease. The original article by J.-H. Shin et al. shows that ROS generated due to ischemia-reperfusion (I/R) injury in bladder muscle reduce muscle tension thereby increasing the risk of lower urinary tract symptoms. The same study illustrated that the xanthine oxidase inhibitor allopurinol could protect the rat urinary bladder from I/R injury and improved the contractile responses of the muscles in longitudinal bladder strips.

All the articles in this special issue focus on redox signaling in the cardiovascular and skeletal muscle system and provide a platform for the researchers to understand the ROS based challenges in these pathologies. The topics covered in this issue set the stage to address many unanswered questions in future.

*Aldrin V. Gomes* *Aldrin V. Gomes* *Namakkal S. Rajasekaran* *Namakkal S. Rajasekaran* *Xinchun Pi* *Xinchun Pi*

